# The risk of disabling, surgery and reoperation in Crohn’s disease – A decision tree-based approach to prognosis

**DOI:** 10.1371/journal.pone.0172165

**Published:** 2017-02-22

**Authors:** Cláudia Camila Dias, Pedro Pereira Rodrigues, Samuel Fernandes, Francisco Portela, Paula Ministro, Diana Martins, Paula Sousa, Paula Lago, Isadora Rosa, Luis Correia, Paula Moura Santos, Fernando Magro

**Affiliations:** 1 Department of Community Medicine, Information and Decision in Health, Faculty of Medicine of the University of Porto, Porto, Portugal; 2 CINTESIS – Center for Health Tecnology and Services Research, Porto, Portugal; 3 Centro Hospitalar Lisboa Norte, Hospital de Santa Maria, Lisboa, Portugal; 4 Gastroenterology Department, Centro Hospitalar e Universitário de Coimbra, Coimbra, Portugal; 5 Gastroenterology Department, Centro Hospitalar Tondela e Viseu, Viseu, Portugal; 6 Gastroenterology Department, Centro Hospitalar do Porto, Porto, Portugal; 7 IPO Lisboa, Lisboa, Portugal; 8 Gastroenterology Department, Hospital São João, Porto, Portugal; 9 Institute of Pharmacology and Therapeutics Faculty of Medicine of the University of Porto, Porto, Portugal; 10 MedInUP - Center for Drug Discovery and Innovative Medicines, University of Porto, Porto, Portugal; Universita degli Studi di Catania, ITALY

## Abstract

**Introduction:**

Crohn’s disease (CD) is a chronic inflammatory bowel disease known to carry a high risk of disabling and many times requiring surgical interventions. This article describes a decision-tree based approach that defines the CD patients’ risk or undergoing disabling events, surgical interventions and reoperations, based on clinical and demographic variables.

**Materials and methods:**

This multicentric study involved 1547 CD patients retrospectively enrolled and divided into two cohorts: a derivation one (80%) and a validation one (20%). Decision trees were built upon applying the CHAIRT algorithm for the selection of variables.

**Results:**

Three-level decision trees were built for the risk of disabling and reoperation, whereas the risk of surgery was described in a two-level one. A receiver operating characteristic (ROC) analysis was performed, and the area under the curves (AUC) Was higher than 70% for all outcomes. The defined risk cut-off values show usefulness for the assessed outcomes: risk levels above 75% for disabling had an odds test positivity of 4.06 [3.50–4.71], whereas risk levels below 34% and 19% excluded surgery and reoperation with an odds test negativity of 0.15 [0.09–0.25] and 0.50 [0.24–1.01], respectively. Overall, patients with B2 or B3 phenotype had a higher proportion of disabling disease and surgery, while patients with later introduction of pharmacological therapeutic (1 months after initial surgery) had a higher proportion of reoperation.

**Conclusions:**

The decision-tree based approach used in this study, with demographic and clinical variables, has shown to be a valid and useful approach to depict such risks of disabling, surgery and reoperation.

## Introduction

Crohn’s disease (CD) is a chronic inflammatory bowel disease for which no definitive treatment has been described. As so, clinicians approach the disease attempting to control the symptoms, avoiding disease complications and improving patients’ quality of life [1]. The most frequent CD complications are related to an uncontrolled inflammation of the bowel, which may cause obstruction and perforation of the small intestine or of the colon, abscess, fistulae and/or intestinal bleeding. The occurrence of these events may require a surgical intervention, which ends up being a common strategy in CD management. In fact, previous studies have reported that around 50% of all CD patients will eventually undergo bowel surgery within 10 years after the diagnosis, whereas 80% will eventually require a surgery throughout the entire disease course [1,2]. Moreover, recurrence is extremely frequent, and the rate of reoperations in previous studies ranged from 40% to 80% [2,3].

As for the concept of disabling, this term was introduced by Beaugerie *et al*. in 2006 [4] and by Loly *et al*. [5] in 2008: both groups evaluated the impact of the disease using clinical and measurable criteria. These studies reported a proportion of disabling disease between 85% and 58%, respectively. Five years after the initial study on this topic, Yang *et al*. [6] presented a new report that settled the proportion of disabling at 80%. However, this last study used a slightly different definition of disabling disease. In fact, given the rapid evolution of disease control strategies, there is currently no consensus on the concept of disabling disease.

The definition of a strong and accurate prognosis model is a key step towards a better disease control and a higher quality of life in CD patients. In this context, this study aimed to unveil the differential impact of several clinical and demographic variables on the CD patients’ risk of surgery, disabling and reoperation, using a decision trees-based strategy.

## Materials and methods

### Derivation and validation cohort

This manuscript describes a multicentric retrospective cohort study including 1547 CD patients recruited from six Portuguese inflammatory bowel disease (IBD) specialist hospitals. Patients were included if 1) had a definitive diagnosis of CD; 2) had at least three years of follow-up; 3) had at least one consultation with a physician involved in this study during 2014 or 2015; and 4) had performed at least an X-ray computed tomography (CT) or a magnetic resonance imaging (MRI) during the follow-up. A hold-out strategy was followed to enable a generalized validation of the prognostic models: the cohort was randomly split into two groups. The first one comprised 80% of patients and constituted the derivation cohort; the held-out remaining 20% of patients were considered to be the validation cohort.

### Clinical and demographic variables

All data was retrieved from GEDII (Grupo de Estudos de Doenças Inflamatórias Intestinais, the Portuguese IBD group) database [7] and included clinical and demographic variables, the dates in which the patients were submitted to bowel surgeries or started immunosuppression, and their classification regarding steroid dependence and refractoriness. The definition of steroid dependence was the inability to reduce steroids below the equivalent of 10 mg per day, prednisolone within 3 months of starting steroids without recurrent active disease, or disease relapse within 3 months of stopping steroids. Steroid resistance was defined as the presence of active disease despite a prednisolone dose of up to 0.75 mg kg^−1^ per day over a period of 4 weeks [8]. The presence and timing of immunosuppressive medication was stratified in four categories: 1) no pharmacological treatment; 2) pharmacological treatment both before and after the first surgery (started within the first month after surgery); 3) pharmacological treatment only after the surgery (starting more than 1 month after the first surgery); and 4) pharmacological treatment only before the first surgery.

### Outcomes analyzed

Three different outcomes were analyzed in this study:

1. disabling disease, defined as a composite endpoint characterized by the presence of at least one of the following events: more than one abdominal surgery or two hospital admissions in the follow-up period; steroid dependence or steroid refractoriness; need for switching the first immunosuppression or anti-TNFα; and the appearance of new clinical events after the index episode (stenosis, anal disease or penetrating disease)[9];
2. surgery, defined as the need for a surgical intervention (abdominal surgery only for CD);
3. reoperation, defined as the need for more than one surgical intervention (abdominal surgery only for CD).

### Statistical analyses

The results of the statistical analysis performed during this study are summarized into decision trees, which are a graphical representation of a possible combination of variables based on specific conditions. It uses a divide-and-conquer strategy to solve a decision problem, which works by dividing a complex problem into simpler problems, recursively applying the same strategy. The different solutions of sub problems are then combined in the form of a tree to produce a solution for the original problem. Each split in the tree (a node) is produced by specifying the percentage of the outcome present in each of the categories of one independent variable (the one that has the highest impact at that level), while the final leaves convey an estimate of the outcome for the subgroup of patients that recursively traversed the tree along that path. Therefore, each path in the tree (from root to leaf) represents an exclusive decision rule associated with an estimate for the outcome. Whereas most decision trees supporting clinical decision problems are expert-based following a deductive reasoning, inductive learning the decision tree from data, *e*.*g*. using recursive partitioning, is a valid method to generate a data-driven decision model [10]. In order to determine the relationship between clinical/demographical factors and outcomes, decision tree classifiers were built from the derivation cohort, applying the CHAID algorithm [11], which is based upon corrected (Bonferroni post-hoc test) chi-squared significant testing. The following variables were analyzed for the outcomes disabling and surgery: gender, smoking habits, age at diagnosis, location disease, behavior, upper tract involvement (L4) and perianal disease. The presence and timing of medical therapeutics were also included when considering the outcome reoperation. The decision tree parameters were validated on the independently held-out validation cohort. The predictive quality of the leaves was evaluated on both cohorts estimating the proportion of the outcome for each of the derived rules.

To assess the discriminative ability of the trees for each outcome, specific cut-off values were chosen after analyzing the ROC curves in the derivation cohort. For disabling disease, a rule-in approach was applied aiming at a high positive predictive value (around 80%). For surgery and reoperation, a rule-out approach was applied aiming at a high negative predictive value (also around 80%). The derived trees (defining exclusive decision rules) were evaluated in both cohorts for the estimation of sensitivity, specificity, accuracy, predictive values, likelihood ratios and post-test odds.

Variables were described through absolute (n) and relative (%) frequencies. The comparison between derivation and validation cohort was made applying a Chi-Square test. All reported p-values were two-sided, for a significance level of 5%. All data were arranged, processed and analyzed with SPSS^®^ v.24.0 (Statistical Package for Social Sciences).

The data collection that was used in this work has been approved by the Portuguese National Committee of Data Protection. This study was conducted according to the principles expressed in the Declaration of Helsinki.

## Results

### Population baseline characteristics and measured outcomes

The derivation cohort consisted of 1245 CD patients, the majority of them female (54%), non-smokers (53%) and diagnosed as young adults (17 to 40 years old, 69%) (Table 1). Disease location and behavior were classified according to the Montreal classifications [12]: 16% had colonic disease and only 12% presented upper tract involvement. Concerning behavior, 46% had a non stricturing/non penetrating phenotype, whereas 26% had perianal disease. Disabling disease occurred in 68% of patients, 47% underwent bowel surgery, and 38% (among the latter) needed reoperation.

**Table 1:** Baseline characteristics and comparison between the derivation and the validation cohorts.

	Derivation (n = 1245)		Validation (n = 302)		p-value
**Gender**					0.409
Male	577	(46%)	132	(44%)	
**Age at diagnosis**					.682
A1 - < = 16 years	138	(11%)	37	(12%)	
A2- 17–40 years	865	(69%)	212	(70%)	
A3- >40 years	242	(19%)	53	(18%)	
**Location**					0.246
L1—Ileon	542	(44%)	120	(40%)	
L2 –Colonic	200	(16%)	44	(15%)	
L3—IleoColonic	503	(40%)	138	(46%)	
**Upper tract involvement (L4)**	152	(12%)	27	(9%)	0.111
**Behaviour**					0.431
B1—Non-Stricturing/non-penetrating	572	(46%)	146	(48%)	
B2—Stricturing	308	(25%)	64	(21%)	
B3—Penetrating	365	(29%)	92	(30%)	
**Perianal disease**	327	(26%)	80	(26%)	0.937
**AZA**					0.403
No AZA	794	(64%)	199	(66%)	
AZA before and after surgery (<1 month)	79	(6%)	16	(5%)	
Aza only after surgery (>1 month)	270	(22%)	70	(23%)	
Aza before surgery	102	(8%)	17	(6%)	
**Anti TNF**					0.835
No anti TNF	943	(76%)	234	(77%)	
anti TNF before and after surgery (<1 month)	44	(4%)	9	(3%)	
anti TNF only after surgery (>1 month)	224	(18%)	53	(18%)	
anti TNF before surgery	34	(3%)	6	(2%)	
**Disabling disease**	849	(68%)	206	(68%)	0.995
**Surgery**	579	(47%)	135	(45%)	0.573
**Reoperation**	220	(38%)	45	(33%)	0.313

### Disabling disease

**Fig 1:**
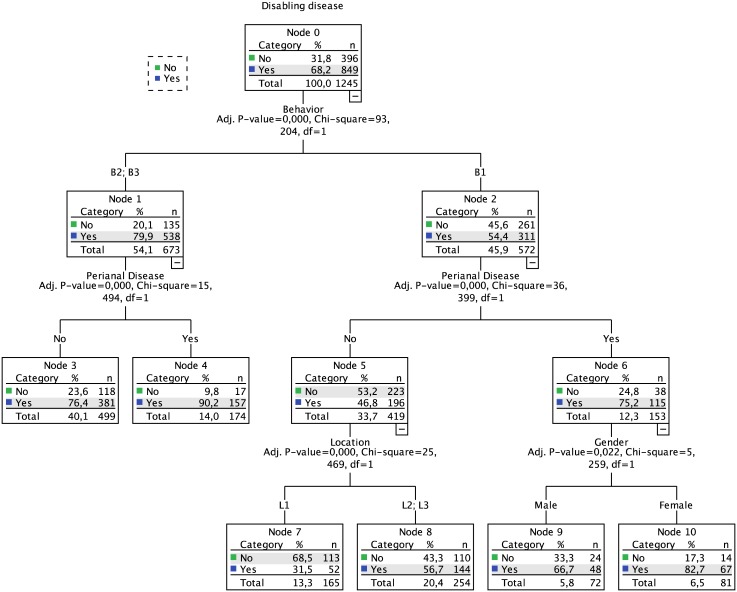
Decision tree for disabling disease.

Disabling disease occurred in 68% of the derivation cohort patients. The induced decision tree, computed from all independent variables with the exception of the presence and timing of pharmacological therapy (as this variable is itself involved in the disabling definition), resulted in a three-level model (Fig 1). The first level was defined by the behavior phenotype, a two-way split that separated the risk for disabling of B1 (54%) apart from that of B2 and B3 phenotypes (80%). The second level consisted in the presence of perianal disease. Location and gender defined the third level for patients that had the B1 phenotype and the absence or presence of perianal disease, respectively. The set of rules defined by this tree can be summarized by the different risk levels (32% to 90%) reported in Table 2. Overall, patients with phenotypes B2 or B3 have a higher risk of disabling disease, while for phenotype B1, gender plays an important role, with female patients having a higher risk than males.

**Table 2:** Decision rules and proportion and confidence intervals of outcomes observed.

**Disabling Disease**		
	Proportion of outcome	For cut-off of 75%
B1 & No Perianal & L1	31.5% [24.4%-38.6%]	46.8% [42.0%-51.6%]
B1 & No Perianal & L2/L3	56.7% [50.6%-62.8%]	
B1 & Perianal & Male	66.7% [55.8%-77.6%]	66.7% [55.8%-77.6%]
B1 & Perianal & Female	82.7% [74.5%-90.9%]	82.7% [74.5%-90.9%]
B2/B3 & No Perianal	76.4% [72.7%-80.1%]	79.9% [76.8%-82.8%]
B2/B3 & Perianal	90.2% [87.8%-94.6%]	
**Surgery**		
	Proportion of outcome	For cut-off of >34%
B1	16.6% [13.5%-19.7%]	16.6% [13.5%-19.7%]
B2 & No L4	72.0% [66.4%-77.6%]	68.2% [62.3%-73.1%]
B2 & L4	51.7% [38.44%-64.6%]	
B3 & Male	81.2% [75.3%-87.1%]	75.1% [70.4%-79.2%]
B3 & Female	69.7% [63.3%-76.2%]	
**Reoperation**		
	Proportion of outcome	For cut-off of > 19%
TNF after surgery (> 1 month) & B2/B3	57.9% [50.9%-64.9%]	52.7% [46.2%-59.1%]
TNF after surgery (> 1 month) & B1	23.5% [9.3%-37.8%]	
TNF (without, before or before and after surgery) & AZA (without or only before surgery) & L1	13.3% [6.6-%-20.0%]	13.3% [6.6%-20.0%]
TNF (without, before or before and after surgery) & AZA (without or only before surgery) & L2/L3	28.9% [18.7%-39.1%]	28.9% [18.7%-39.1%]
TNF (without, before and before or after surgery) & (AZA after or before and after surgery)	37.0% [30.3%-44.3%]	37.0% [18.7%-39.1%]

Logical operators: & (AND); White—< = 10%; green– 11–19%; Yellow– 20%-49%; Orange– 50%-74% and red> = 75%

### Surgery

**Fig 2:**
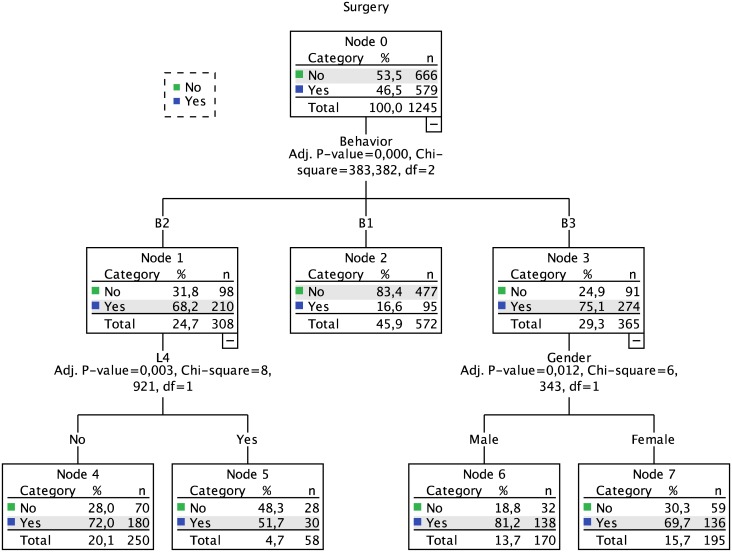
Decision tree for surgery.

The outcome surgery affected 47% of the derivation cohort patients. The induced decision tree, computed using the same variables as those used for the disabling decision tree, resulted in a two-level model (Fig 2). As for the disabling, the first level was defined by disease behavior, although in this case a three-way split separated the surgery risk of all phenotypes: 16% for B1, 68% for B2, and 75% for B3. The second level of the model included information on upper track involvement (L4) (for patients with the B2 phenotype) and gender (for patients with the B3 phenotype). The set of rules hereby defined represent different risk levels (17% to 81%), which are depicted in Table 2. Globally, patients with a B3 phenotype have a higher risk of surgery than those with a B1 behavior, and this risk is further aggravated in male B3 patients.

### Reoperation

**Fig 3:**
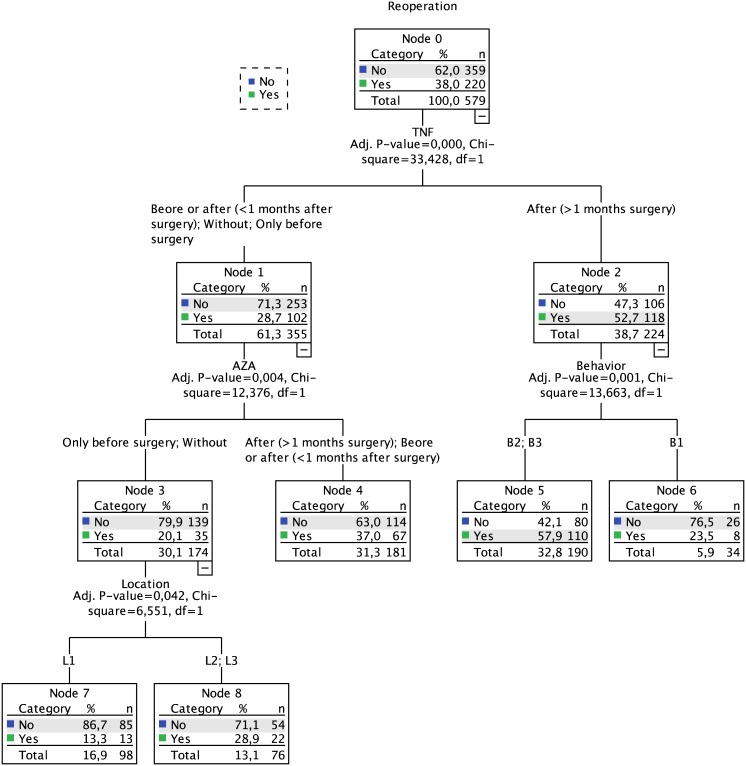
Decision tree for reoperation.

The rate of reoperation was defined among those patients that underwent bowel surgery: 38% required additional surgical interventions. The induced decision tree, computed using all variables described before and including the timing and presence of pharmacological therapeutics, resulted in a three-level model of variables (Fig 3). The first level was defined by the presence and timing of the anti-TNF introduction, separating those that started anti-TNF more than one month after surgery (53% of reoperation risk) from all the others (29% of reoperation risk). The second level included behavior for the former (stratified in B1 *vs*. B2/B3) and presence and timing of AZA introduction for the later (discriminating between patients that have either never been medicated or been medicated only before surgery from the remaining). The third level encompassed the disease location, separating L1 from L2 and L3. The defined set of rules resulted in different risk levels (13% to 58%) that are listed in Table 2. Overall, patients with a later introduction of pharmacological intervention (1 month after initial surgery) had a worse outcome, i.e, they have a higher probability of undergoing more than one surgery during the disease course.

### Model validation

The validation cohort consisted of 302 patients and was similar to the derivation cohort concerning the frequency of the analyzed variables and outcomes (Table 1). The risk of each outcome following the decision rules extracted from the trees in the derivation and validation cohorts (S1 Fig). The proportion of the outcomes is similar in both cohorts, and their confidence intervals are overlapping, therefore attesting the robustness of the decision rules.

Receiver operating characteristic (ROC) analysis were performed independently for the derivation and validation cohorts and the respective AUC values were similar in and had overlapping 95% CI for disabling and surgery, but not for reoperation (S2 Fig). Moreover, the AUCs were rather heterogeneous for the different outcomes: the derivation cohort presented an AUC of 72% for disabling, 80% for surgery and 69% for reoperation.

The derivation cohort ROC curves were used to establish cut-offs able to assess the likelihood of the occurrence of each outcome. Positive results were defined for risk levels above 75% concerning disabling disease, above 34% concerning surgery, and above 19% concerning reoperation. These cut-offs enabled the computation of a simplified set of rules that are listed in Table 2. The performance parameters of the chosen cut-offs considering each outcome are depicted in Table 3 for the derivation and the validation cohorts. Most of the 95% CI overlapped between both cohorts, once again validating the initial model. Overall, the application of the cut-offs to the validation cohort resulted in 81% [74%-86%] PPV for disabling disease, 87% [80%-92%] NPV for surgery, and 67% [57%-75%] for reoperation.

**Table 3:** Performance of risk matrix in derivation and validation cohort for disabling disease and reoperation (% [CI 95%]).

	Disabling >75%		Surgery >34%		Reoperation >19%	
	Derivation	Validation	Derivation	Validation	Derivation	Validation
Sens	71 [68–74]	67 [61–74]	**84 [80–86]**	**86 [78–91]**	**94 [89–97]**	**87 [73–94]**
Spec	**62 [57–74]**	**65 [55–75]**	72 [68–75]	76 [69–82]	24 [19–28]	13 [7–23]
PPV	**80 [77–83]**	**81 [74–86]**	72 [68–75]	74 [66–81]	43 [39–48]	33 [8–21]
NPV	50 [45–55]	48 [40–58]	**83 [80–86]**	**87 [80–92]**	**87 [78–92]**	**67 [57–75]**
LR+	1.89 [1.66–2.16]	1.97 [1.47–2.65]	2.94 [2.60–3.34]	3.59 [2.71–4.74]	1.23 [1.15–1.32]	1.00 [0.67–1.15]
LR-	0.46 [0.41–0.51]	0.49 [0.40–0.60]	0.23 [0.19–0.28]	0.19 [0.12–0.28]	0.25 [0.14–0.43]	1.00 [0.41–2.43]
Odds post test+	**4.06 [3.50–4.71]**	**4.24 [3.09–5.81]**	2.56 [2.25–2.92]	2.90 [2.18–3.85]	0.75 [0.66–0.86]	0.5 [0.37–0.67]
Odds post test-	0.99 [0.90–1.08]	1.05 [0.87–1.26]	**0.20 [0.16–0.24]**	**0.15 [0.09–0.23]**	**0.15 [0.09–0.25]**	**0.5 [0.24–1.01]**

Sens: Sensibility; Spec: Specifity; PPV: Positive Predictive value; NPV: Negative Predictive value; LR+: Positive Likelihood ratio: LR-: Negative Likelihood ratio

## Discussion

Given the impact and frequency of recurrences among CD patients, the development of prognostic models is a cornerstone to guide physicians in their therapeutic choices and to improve patients’ well-being. The most important characteristics of these models are their user-friendliness and readability, allowing a fast and effortless readout during patient encounters or upon the need to decide on a therapeutic approach.

This cohort presented a disabling rate of 68%, a similar value to that depicted in previous studies of different Portuguese cohorts [9,13]. However, other authors have reported higher disabling rates [4–6]. This difference is likely due to the fact that the disabling definition used in this study is stricter than that used in previous ones, namely by excluding the need of immunosuppression or anti-TNF as criteria. In our opinion, the introduction of pharmacological therapy not qualify as disabling, given the top-down and accelerated step-up strategies currently followed to approach CD.

Surgery, on its turn, affected 47% of the patients in the derivation cohort. This value is lower than that presented by Bernal *et al*. [2], which could be related to the fact that the cohort analyzed in that study was composed of older patients (data collected since 1955). The rapid evolution of CD therapeutics and the current strategies used to approach the disease, together with the fact that our cohort included patients that have been more recently diagnosed, explains our lower surgery rate. Moreover, a recent meta-analysis has reported a 47% risk of surgery within 10 years after diagnosis [14], thereby supporting the results described here. Reoperation, on the other hand, affected 38% of the patients who underwent a first surgery, a rate similar to that presented in a recent meta-analysis that settled the 10-years risk of reoperation at 33% [15].

The results from this study are depicted in three decision trees that represent the risk for each of the outcomes described above taking into specific combinations of clinical and demographic variables. These decision trees were validated in an independent cohort by; a) comparing the proportion of the outcome in each derived rule; and b) comparing the diagnostic performance parameters using specific risk cut-off levels. The proportion of each outcome following the decision rules in the different cohorts was similar. Moreover, the comparison of the diagnostic performance parameters revealed that the decision trees had a good prognostic ability of concerning disabling disease and surgery: the validation cohort had a positive post-test odds for disabling disease of 4.24 [3.09–5.81], and a negative post-test odds for surgery of 0.15 [0.09–0.23]. Reoperation, on the other hand, appeared to a harder outcome to predict, presenting a less favorable performance among the validation cohort patients. This might indicate that other factors—besides those that have been considered—need to be included in the model. Nevertheless, the negative post-test odds of the validation cohort were 0.5 [0.24–1.01], and therefore this model might still be useful to detect patients with a lower risk of reoperation.

The variables used in the decision tree were chosen by applying the CHAID algorithm, which is similar to the chi-square with Bonferroni correction post hoc test. The final selection of variables was the same as that used in previous studies that employed different selection methods, therefore attesting the robustness of the computed decision trees [2,4–6,16–18]. The decision tree analysis has a some advantages over others that are more widely used *(e*.*g*. logistic regression). An undeniable strength of this method is its graphical representation, which allows a quick and intuitive reading. On the other hand, decision trees are rather flexible in the way that they do not assume any data distribution. Another advantage is the attribute selection, which restricts the variables in the model to those that are non-redundant. The interpretability of the trees is also one their strong points—complex decisions can be approximated by simple or local decisions. Finally, decision trees allow an easy comparison of patients’ subgroups, as decision rules can be created directly from the tree. Overall, patients with B2 or B3 phenotype had a higher proportion of disabling disease and surgery, while patients with later introduction of pharmacological interventions (one month after initial surgery) had a higher proportion of reoperation. Although analyzed in a retrospective-fashion and using retrospectively-defined outcomes, this study presents an analysis of a large multicentric cohort formally validated by the application of the derived results in a validation cohort.

In conclusion, we have shown that variables such as disease behavior, upper gastrointestinal involvement, gender, perianal disease, location and medical therapeutics affect the risk of disabling disease, surgery and reoperation in CD patients. Moreover, these variables impact the aforementioned outcomes at different levels, having different weights in sub-groups of patients with different variables’ combinations. Our results are represented in three graphical and user-friendly bedside tools that can be used by the physicians to assess the risk of disabling, surgery and reoperation in CD patients, therefore supporting the decision making process regarding therapeutic strategies. A disabling risk above 75% allows the prediction of disabling events with a PPV of 81% and an odds post-test positivity of 4.24, whereas a surgery risk inferior to 34% allows the exclusion of future surgeries with a NPV of 87% and an odds post-test negativity of 0.15. The reoperation was the hardest outcome to predict, although a risk below 19% could be useful for excluding future events (NPV: 87 and odds post-test negativity: 0.5).

## Supporting information

S1 Fig Proportion and 95% confidence interval of outcome in derivation and validation cohort. (JPG)

S2 Fig AUC. (PNG)
