# Supplementary figures and images for: The risk of disabling, surgery and reoperation in Crohn’s disease – A decision tree-based approach to prognosis
Source: PLoS One. 2017 Feb 22;12(2):e0172165. doi: 10.1371/journal.pone.0172165 (PMC5321294; doi:10.1371/journal.pone.0172165)

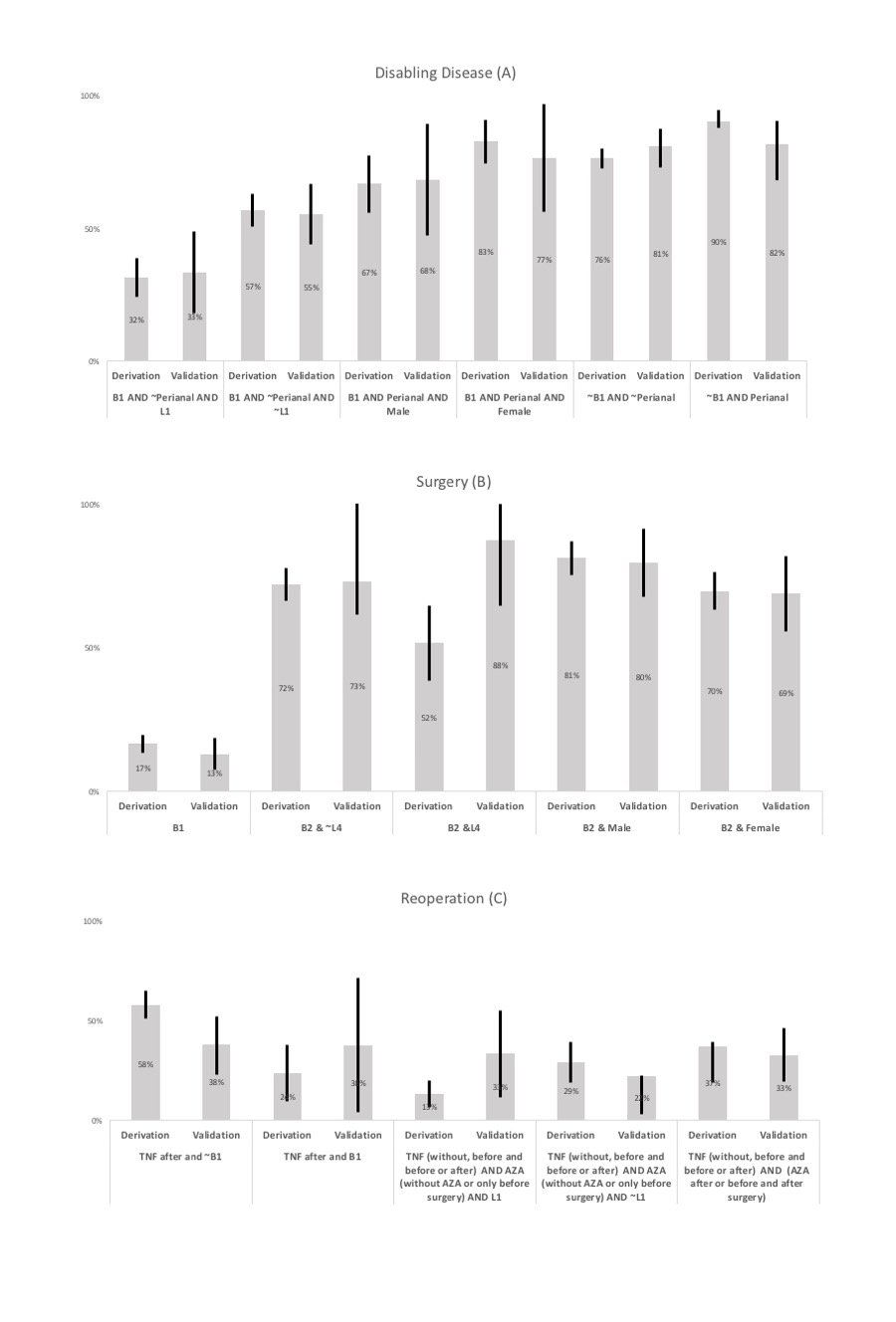

Supplement: S1 Fig — (JPG) [file pone.0172165.s001.jpg]

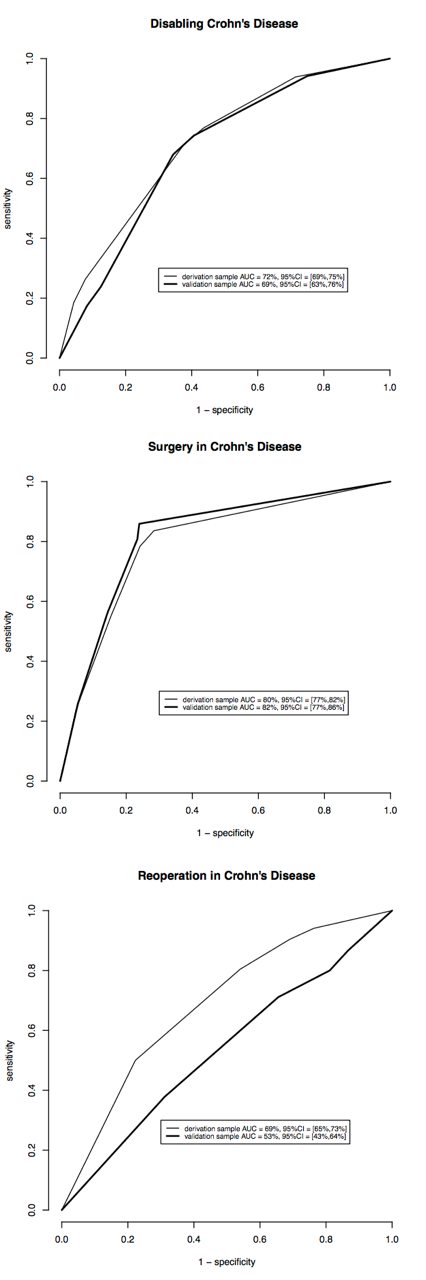

Supplement: S2 Fig — (PNG) [file pone.0172165.s002.png]
